# Supplementary material for: A novel proneural function of Asense is integrated with the sequential actions of Delta-Notch, L’sc and Su(H) to promote the neuroepithelial to neuroblast transition
Source: PLoS Genet. 2023 Oct 23;19(10):e1010991. doi: 10.1371/journal.pgen.1010991 (PMC10621995; doi:10.1371/journal.pgen.1010991)
Supplement: S1 Fig — Surface (A1, B1) and deep layer confocal sections (A2, B2) showing GFP expression in the OPC of c820-Gal4/UAS-dGFP and c855a-Gal4/UAS-dGFP larval brains. In the c820>GFP sample, GFP is present in the transition cell (L’sc+) and in medial NE cells, while the c855a>GFP specimen exhibits strong GFP labeling in NE cells but weak or absent in transition cells. (PDF) [file pgen.1010991.s001.pdf]

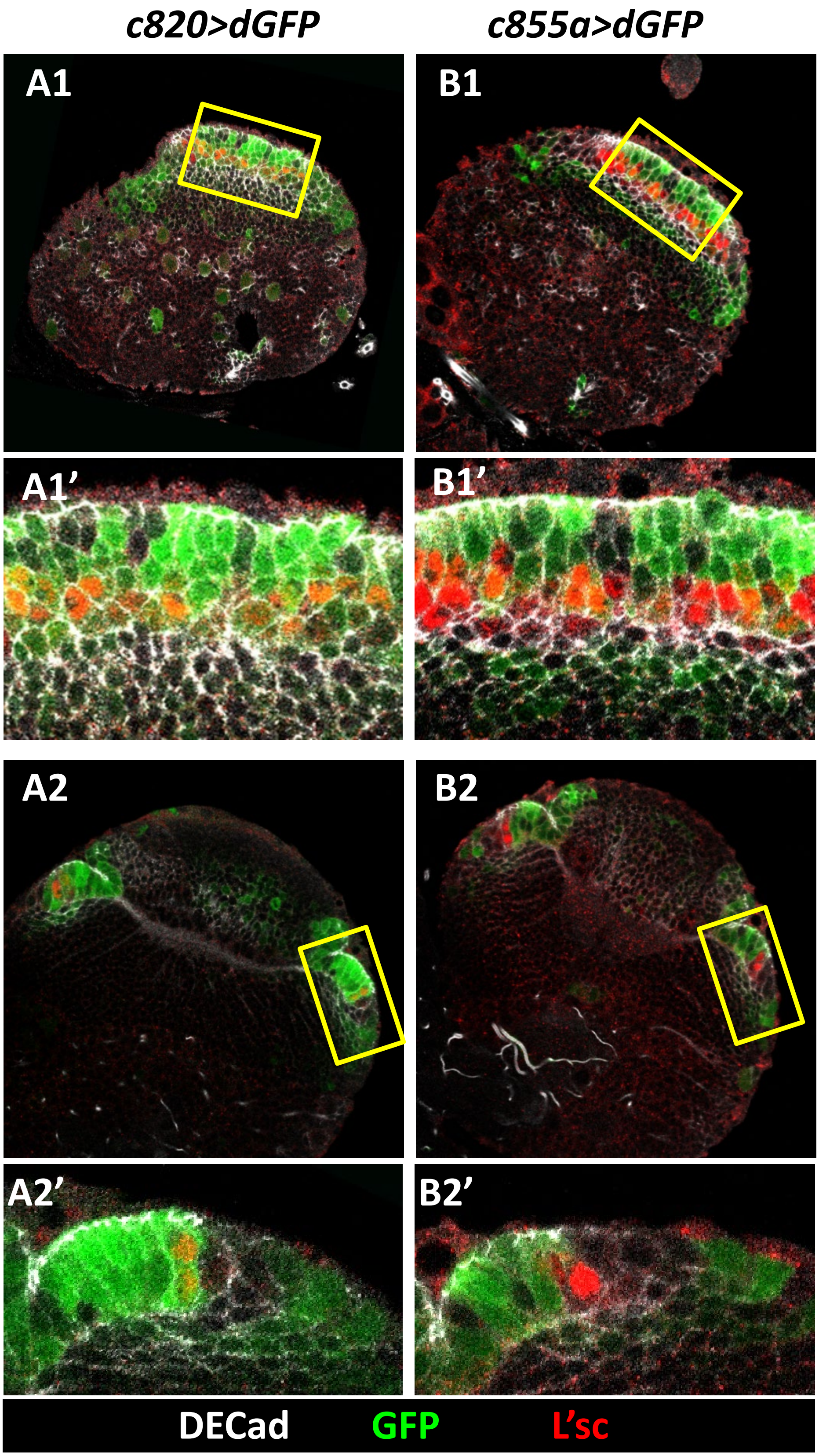

S1 Fig

S1 Fig. Expression patterns of Gal4 drivers at the NE-NB transition. Surface (**A1, B1**) and deep layer confocal sections (**A2, B2**) showing GFP expression in the OPC of *c820-Gal4/UAS-dGFP* and *c855a-Gal4/UAS-dGFP* larval brains. In the *c820>GFP* sample*,* GFP is present in the transition cell (L'sc^{+}) and in medial NE cells, while the *c855a>GFP* specimen exhibits strong GFP labeling in NE cells but weak or absent in transition cells.
