# Supplementary material for: A novel proneural function of Asense is integrated with the sequential actions of Delta-Notch, L’sc and Su(H) to promote the neuroepithelial to neuroblast transition
Source: PLoS Genet. 2023 Oct 23;19(10):e1010991. doi: 10.1371/journal.pgen.1010991 (PMC10621995; doi:10.1371/journal.pgen.1010991)
Supplement: S2 Fig — GFP expression in the OPC (deep layer) of c855a-Gal4/UAS-dGFP larval brains incubated at 17°C all along the larval developmental time (A) or at 17°C until mid third instar stage followed by 24h at 30°C (B) (see Materials and Methods). C,D, Confocal images taken close to the surface of the OPC of control and c855a-Gal4/UAS-ase larval brains incubated at 17°C. Only 3 out of the 7 c855a-Gal4/UAS-ase analyzed brains presented a very subtle phenotype consisting on having 1 Dpn+ cell (arrowhead) within the OPC NE. E. Deep layer confocal image of the OPC of a c855a-Gal4/UAS-L’sc larval brain incubated at 30°C during the last 12h of development (see Materials and Methods). Note that the ectopic L’sc+ cells driven by c855a-Gal4 in the NE (white elipse) have stronger labeling than the endogenous (transition) L’sc+ cells (green elipse). (PDF) [file pgen.1010991.s002.pdf]

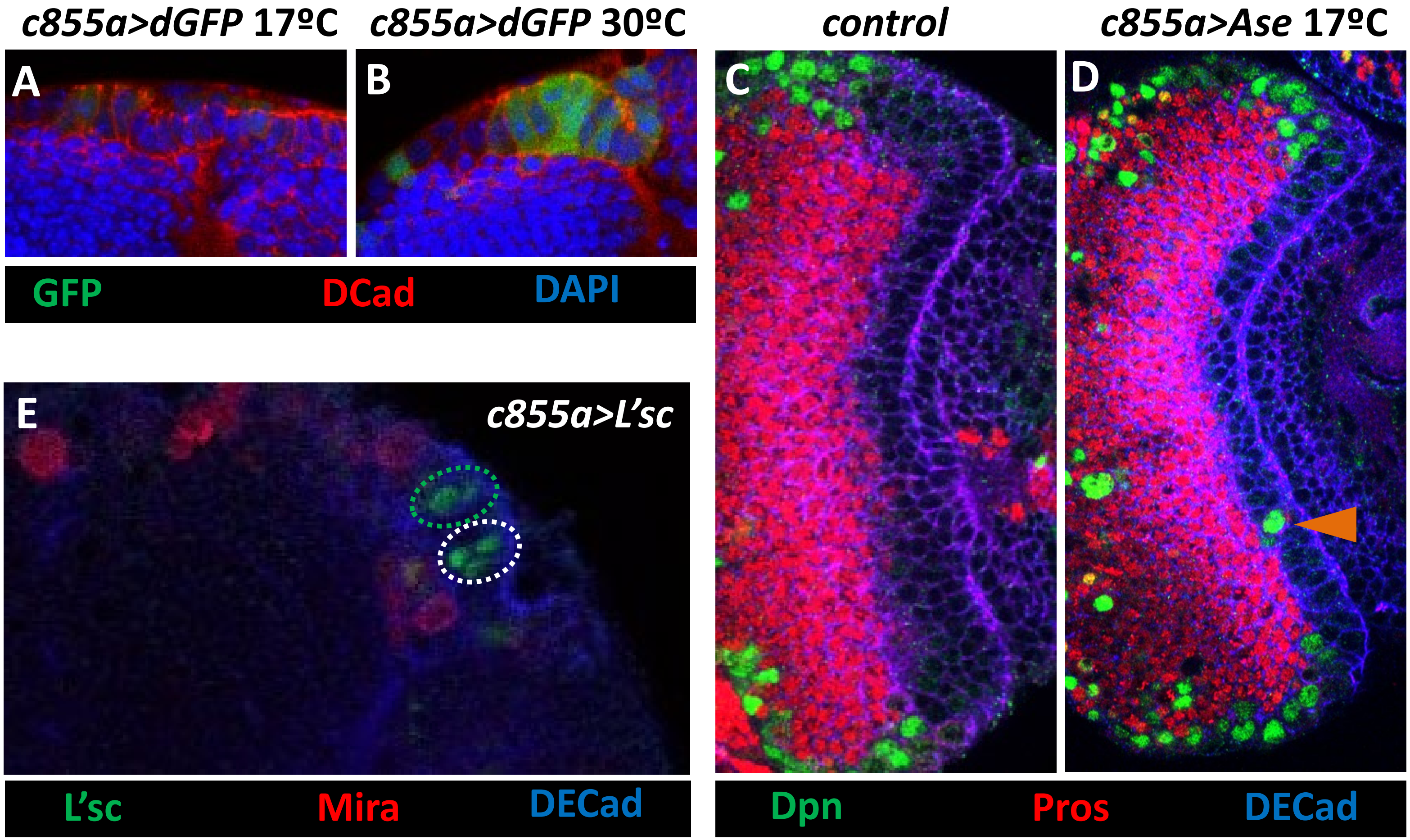

S2 Fig. Effect of temperature on the expression of GFP, Ase, and L'sc induced by *c855a-Gal4*. GFP expression in the OPC (deep layer) of *c855a-Gal4/UAS-dGFP* larval brains incubated at 17ºC all along the larval developmental time (**A**) or at 17ºC until mid third instar stage followed by 24h at 30ºC (**B**) (see Materials and Methods). **C,D**, Confocal images taken close to the surface of the OPC of control and *c855a-Gal4/UAS-ase* larval brains incubated at 17ºC. Only 3 out of the 7 *c855a-Gal4/UAS-ase* analyzed brains presented a very subtle phenotype consisting on having 1 Dpn+ cell (arrowhead) within the OPC NE. **E**. Deep layer confocal image of the OPC of a *c855a-Gal4/UAS-L'sc* larval brain incubated at 30ºC during the last 12h of development (see Materials and Methods). Note that the ectopic L'sc^{+} cells driven by c855-Gal4 in the NE (white elipse) have stronger labeling than the endogenous (transition) L'sc+ cells (green elipse).
