# Supplementary material for: A novel proneural function of Asense is integrated with the sequential actions of Delta-Notch, L’sc and Su(H) to promote the neuroepithelial to neuroblast transition
Source: PLoS Genet. 2023 Oct 23;19(10):e1010991. doi: 10.1371/journal.pgen.1010991 (PMC10621995; doi:10.1371/journal.pgen.1010991)
Supplement: S3 Fig — A, B. Deep layer confocal section of control and c820 >ase-RNAi samples. A1, B1. High magnification views of the framed areas showing that the c820 >ase-RNAi sample lacks peak of ase cells compared to the control (green arrowhead) and exhibits weakly labeled Ase+ cells co-expressing Mira and DECad (read/white arrowhead). C. Quantification of the number of peak of Ase cells along 20 μm of OPC Z axis in 10 larval brains of control (c820-Gal4) and c820>ase-RNAi larvae. Differences are statistically significant (Mann-Whitney Rank Sum Test, P<0.001) D. Quantification of Mira/Cad co-expressing cells in control and c820>ase-RNAi (13 OLs). Statistical significance was assessed with Mann-Whitney Rank Sum Test (P<0.001). (PDF) [file pgen.1010991.s003.pdf]

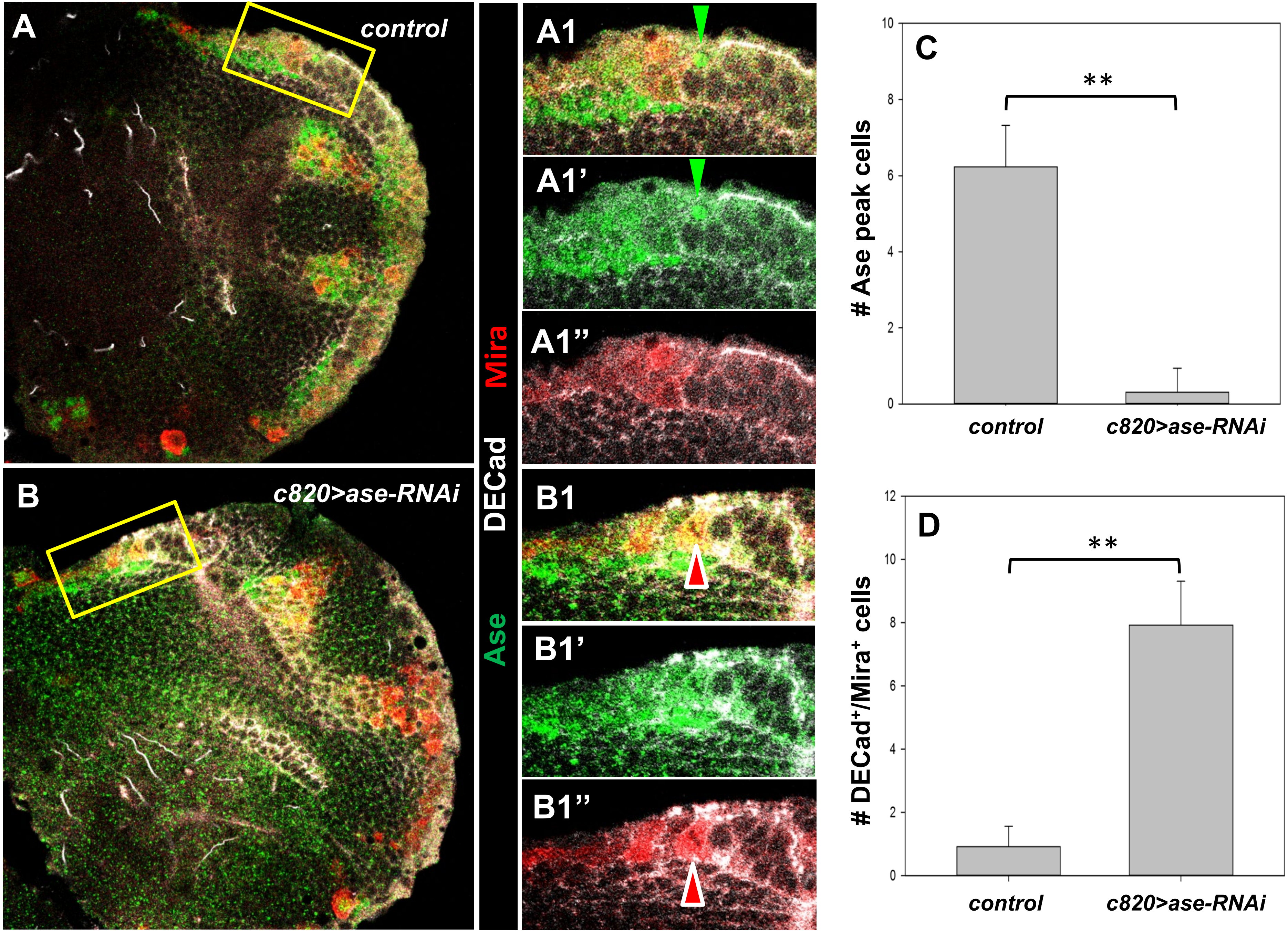
A
control
B
c820>ase-RNAi
A1
A1'
A1''
B1
B1'
B1''
Ase
DECad
Mira
C
Ase peak cells
**
control
c820>ase-RNAi
D
DECad+/Mira+ cells
**
control
c820>ase-RNAi

S3 Fig

S3 Fig. Alterations of the NE-NB transition by *ase RNAi* driven by *c820-Gal4*. A, B. Deep layer confocal section of control and *c820 >ase-RNAi* samples. **A1, B1**. High magnification views of the framed areas showing that the *c820 >ase-RNAi* sample lacks peak of *ase* cells compared to the control (green arrowhead) and exhibits weakly labeled Ase+ cells co-expressing Mira and DECad (read/white arrowhead). **C**. Quantification of the number of peak of Ase cells along 20 μm of OPC Z axis in 10 larval brains of control (*c820-Gal4*) and *c820>ase-RNAi* larvae. Differences are statistically significant (Mann-Whitney Rank Sum Test, P<0.001) **D.** Quantification of Mira/Cad co-expressing cells in control and *c820>ase-RNAi* (13 OLs). Statistical significance was assessed with Mann-Whitney Rank Sum Test (P <0.001).
