# Supplementary material for: A novel proneural function of Asense is integrated with the sequential actions of Delta-Notch, L’sc and Su(H) to promote the neuroepithelial to neuroblast transition
Source: PLoS Genet. 2023 Oct 23;19(10):e1010991. doi: 10.1371/journal.pgen.1010991 (PMC10621995; doi:10.1371/journal.pgen.1010991)
Supplement: S4 Fig — Confocal images taken close to the surface (A,C) or in deep layers (B,D) of the OPC of control (c855a-Gal4) and c855a-Gal4/UAS ase larval brains after a 8h induction. Note the presence of Mira+ cells intermingled in the NE (yellow arrowheads) of the c855>ase specimen compared to the control. E-H. Equivalent images taken from control (c855a Gal4) and c855a Gal4/UAS l’sc larval brains after a 12 h induction. Note that despite the large number of L’sc+ cells (green arrowheads) inside the medulla NE (left side) of the c855>ase specimen there are no Mira+ cells on it. In contrast, there are several ectopic Mira+ cells in the lamina NE (right side, orange arrowheads). (PDF) [file pgen.1010991.s004.pdf]

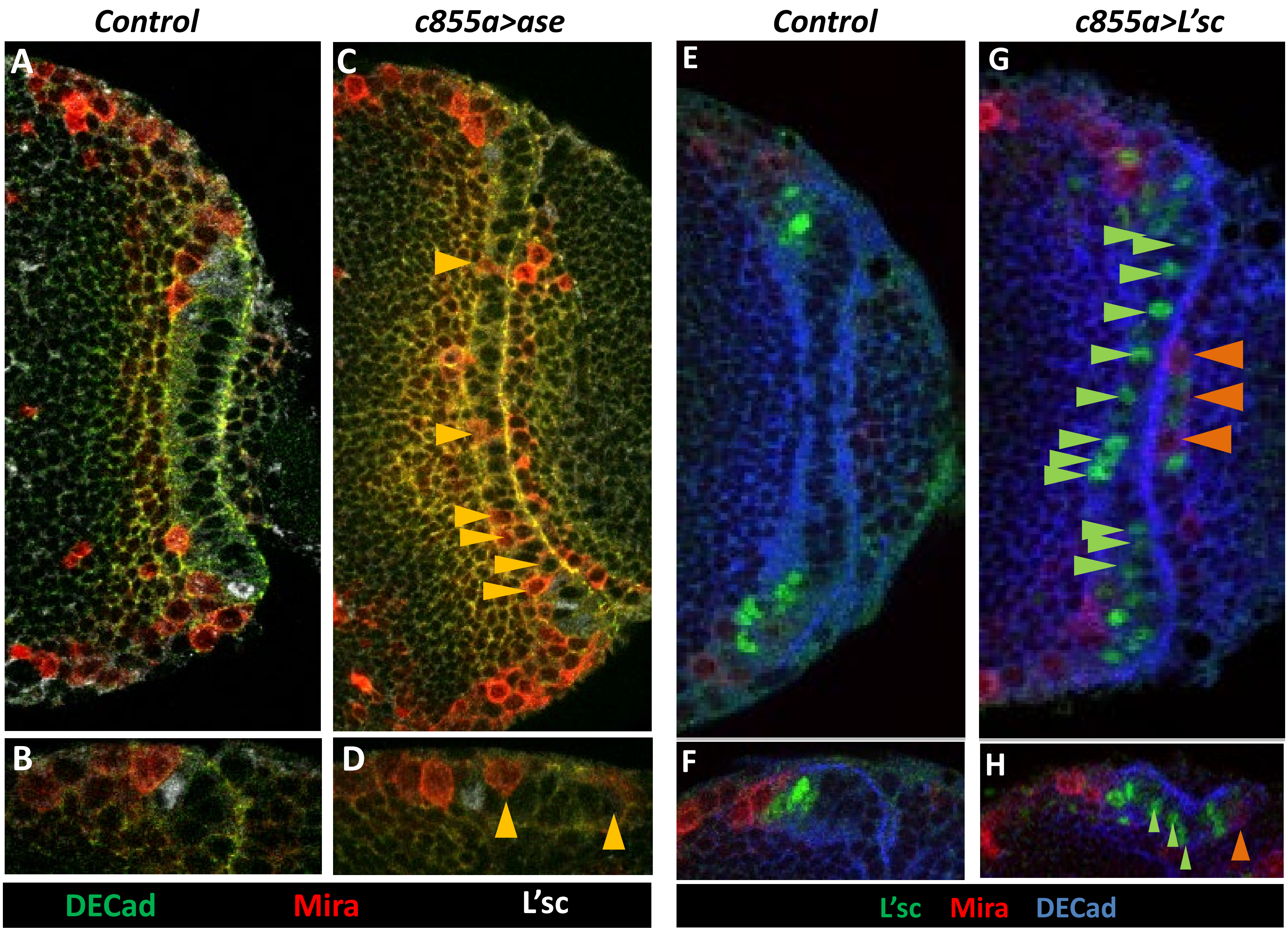
Control
c855a>ase
Control
c855a>L'sc
A
C
E
G
B
D
F
H
DECad
Mira
L'sc
L'sc
Mira
DECad

S4 Fig

S4 Fig. **Differential effects of Ase and L'sc GoFs on the expression of Mira in the NE**. Confocal images taken close to the surface (**A,C**) or in deep layers (**B,D**) of the OPC of control (*c855a Gal4*) and *c855a Gal4/UAS ase* larval brains after a 8h induction. Note the presence of Mira^{+} cells intermingled in the NE (yellow arrowheads) of the *c855>ase* specimen compared to the control. **E-H**. Equivalent images taken from from control (*c855a Gal4*) and *c855a Gal4/UAS l'sc* larval brains after a 12 h induction. Note that despite the large number of L'sc^{+} cells (green arrowheads) inside the medulla NE (left side) of the *c855>ase* specimen there are no Mira+ cells on it. In contrast, there are several ectopic Mira+ cells in the lamina NE (right side, orange arrowheads).
