# Supplementary material for: A novel proneural function of Asense is integrated with the sequential actions of Delta-Notch, L’sc and Su(H) to promote the neuroepithelial to neuroblast transition
Source: PLoS Genet. 2023 Oct 23;19(10):e1010991. doi: 10.1371/journal.pgen.1010991 (PMC10621995; doi:10.1371/journal.pgen.1010991)
Supplement: S5 Fig — Confocal images taken close to the surface (A,B) of the OPC of control (c855a Gal4) and c855a-Gal4/UAS-Dl-DN larval brains after a 9 h induction. Note the lower Notch labeling in medial edge of the NE in the control specimen (B, white dotted line area) and the fall of Notch expression in the whole NE (green dotted line area) of the c855a>Dl-DN sample (B’), C,D. Confocal images taken in deep layers of the OPC of control (c855a-Gal4) and c855a-Gal4/UAS-Dl-DN larval brains after a 12 h induction. Note that in the c855a>Dl-DN sample, the first cell medial to the NE exhibits strong Mira and weak Ase labeling (green arrow) in contrast to the stoong peak of Ase expression in the control sample (red arrow). E Quantification of the number of peak of Ase cells along 20 μm of OPC Z axis in control (c855a-Gal4) and c855a-Gal4/UAS-Dl-DN larval brains after a 8h induction. Differences are statistically significant (Mann-Whitney Rank Sum Test, P = 0.002). F,G. Confocal images taken close to the surface of the OPC of control and, c855a-Gal4/UAS-L’sc larval brains after a 12h induction. The NE of the c855a>L’sc specimen is almost identical to the control sample except for the presence of a single Ase+ cell (arrow). (PDF) [file pgen.1010991.s005.pdf]

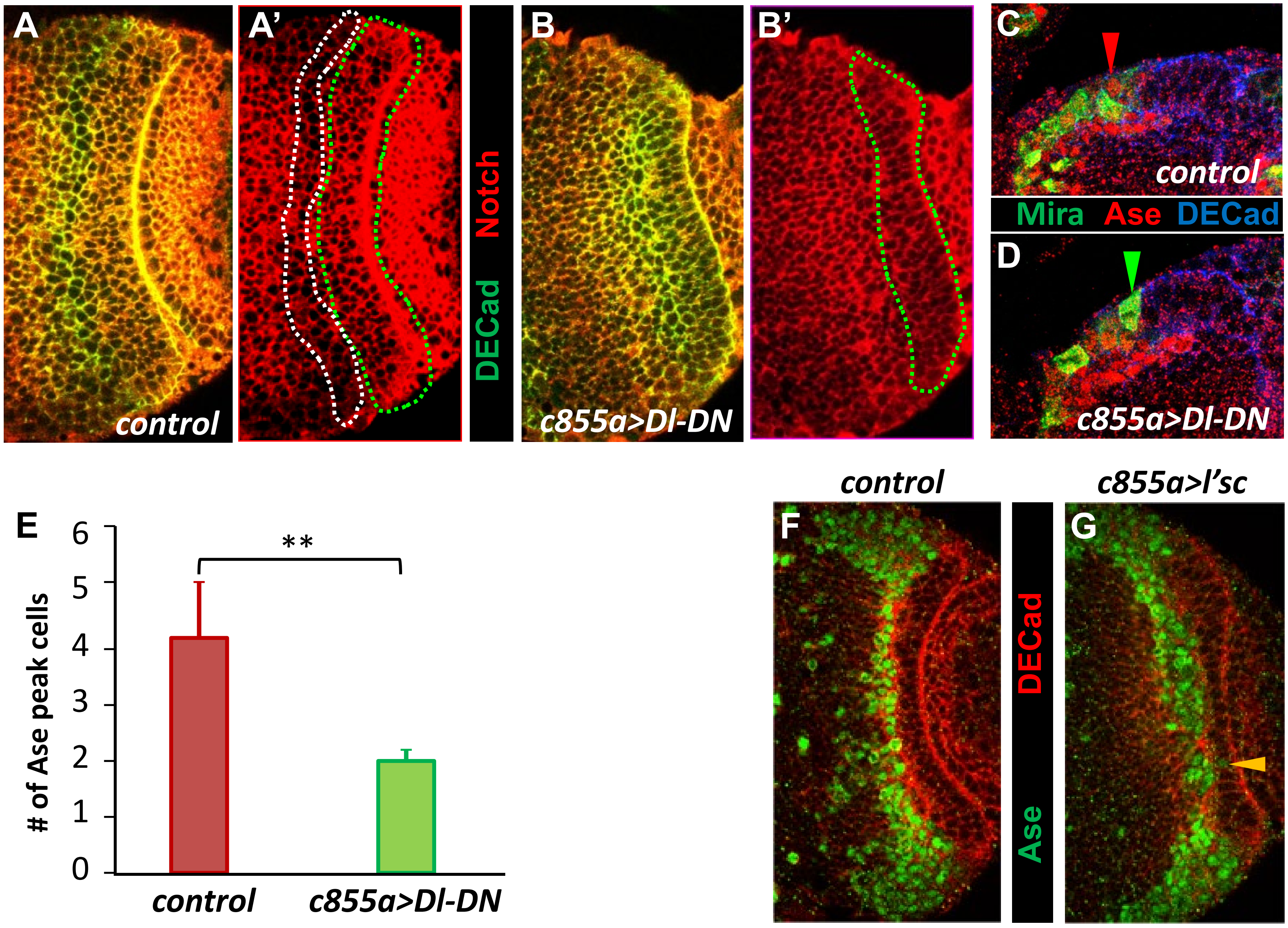
A
control
A'
Notch
DECad
B
c855a>Dl-DN
B'
C
control
Mira Ase DECad
D
c855a>Dl-DN
E
# of Ase peak cells
**
0
1
2
3
4
5
6
control
c855a>Dl-DN
control
c855a>l'sc
F
G
DECad
Ase

S5 Fig

S5 Fig. L'sc misexpression and Notch down regulation do not induce Ase expression in the NE. Confocal images taken close to the surface (**A,B**) of the OPC of control (*c855a Gal4*) and *c855a-Gal4/UAS-Dl-DN* larval brains after a 9 h induction. Note the lower Notch labeling in medial edge of the NE in the control specimen (B, white dotted line area) and the fall of Notch expression in the whole NE (green dotted line area) of the *c855a>Dl-DN* sample (B'), **C,D.** Confocal images taken in deep layers of the OPC of control (*c855a-Gal4*) and *c855a-Gal4/UAS-Dl-DN* larval brains after a 12 h induction. Note that in the *c855a>Dl-DN* sample, the first cell medial to the NE exhibits strong Mira and weak Ase labeling (green arrow) in contrast to the stoong peak of Ase expression in the control sample (red arrow). **E** Quantification of the number of peak of Ase cells along 20 µm of OPC Z axis in control (*c855a Gal4*) and *c855a-Gal4/UAS-Dl-DN* larval brains after a 8h induction. Differences are statistically significant (Mann-Whitney Rank Sum Test, P=0.002). **F,G**. Confocal images taken close to the surface of the OPC of control and, *c855a-Gal4/UAS-L'sc* larval brains after a 12h induction. The NE of the *c855a>L'sc* specimen is almost identical to the control sample except for the presence of a single Ase+ cell (arrow).
