# Supplementary material for: A novel proneural function of Asense is integrated with the sequential actions of Delta-Notch, L’sc and Su(H) to promote the neuroepithelial to neuroblast transition
Source: PLoS Genet. 2023 Oct 23;19(10):e1010991. doi: 10.1371/journal.pgen.1010991 (PMC10621995; doi:10.1371/journal.pgen.1010991)
Supplement: S6 Fig — Effects on the NE-NB transition. Close to surface confocal image of the OPC of control and c855a>N RNAi samples after induction for 9 and 36h. Notice that after 9h induction there is no apparent decrease in Notch labeling compared to the control (A’, B’). In contrast, after 36h induction Notch labeling is very weak (C’). Numerous Dpn/DECad co-expressing cells (arrows) can be detected in the c855a>N RNAi sample after 36h induction (C) (PDF) [file pgen.1010991.s006.pdf]

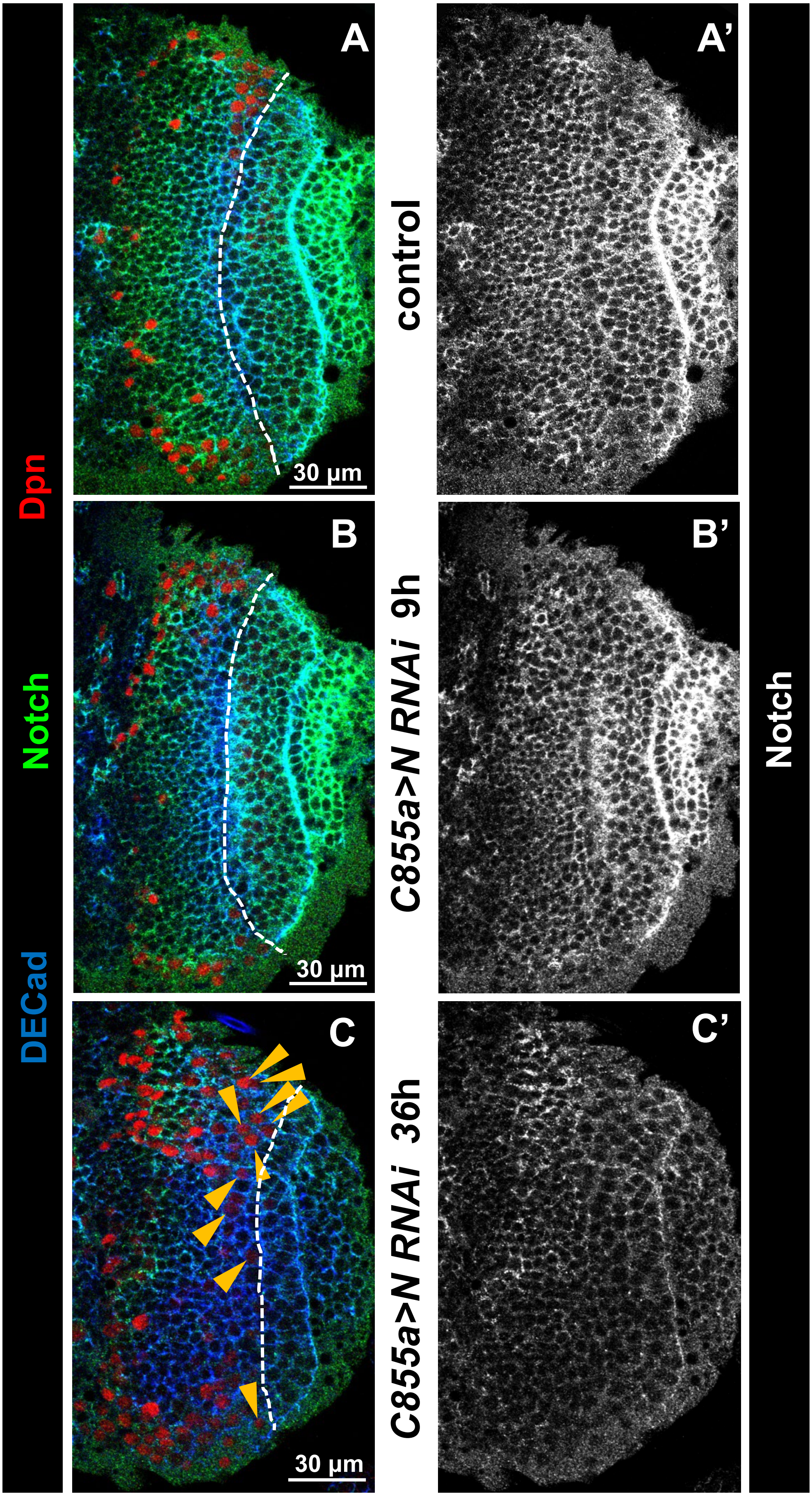

A
A’
B
B’
C
C’
30 µm
30 µm
30 µm
control
C855a>N RNAi 9h
C855a>N RNAi 36h
Dpn
Notch
DECad
Notch

S6 Fig 6

S6 Fig. Timing of Notch protein downregulation induced by *Notch RNAi* driven by *c855a-Gal4*. Effects on the NE-NB transition. Close to surface confocal image of the OPC of control and *c855a>N RNAi* samples after induction for 9 and 36h. Notice that after 9h induction there is no apparent decrease in Notch labeling compared to the control (**A', B'**). In contrast, after 36h induction Notch labeling is very weak (**C'**). Numerous Dpn/DECad co-expressing cells (arrows) can be detected in the *c855a>N RNAi* sample after 36h induction (**C**)
