# Supplementary material for: A novel proneural function of Asense is integrated with the sequential actions of Delta-Notch, L’sc and Su(H) to promote the neuroepithelial to neuroblast transition
Source: PLoS Genet. 2023 Oct 23;19(10):e1010991. doi: 10.1371/journal.pgen.1010991 (PMC10621995; doi:10.1371/journal.pgen.1010991)
Supplement: S7 Fig — Confocal images taken at deep layers of the OPC of control (c820-Gal4) and c820-Gal4/UAS-Dpn-RNAi larval brains after a 24h induction. Note that despite the complete suppression of Dpn immunostaining there is no increase in Ase labeling in the NBs of the c820>Dpn-RNAi relative to the NBs of the control sample and to its own Ase peak cell (arrowhead). (PDF) [file pgen.1010991.s007.pdf]

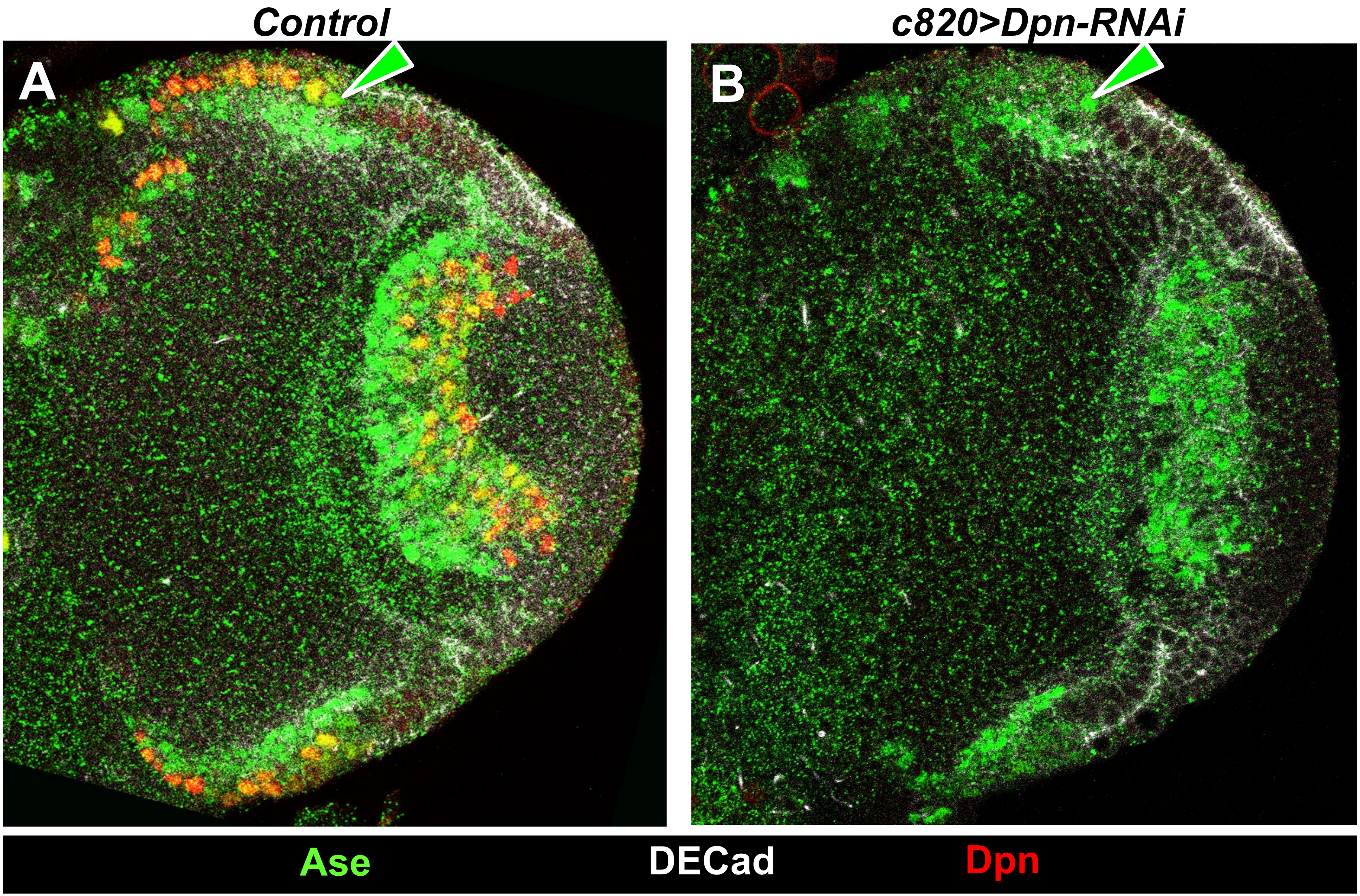

S7 Fig. The downregulation of *Dpn* in NBs does not modify Ase expression. Confocal images taken at deep layers of the OPC of control (*c820 Gal4*) and *c820 Gal4/UAS Dpn-RNAi* larval brains after a 24h induction. Note that despite the complete suppression of Dpn immunostaining there is no increase in Ase labeling in the NBs of the *c820>Dpn-RNAi* relative to the NBs of the control sample and to its own Ase peak cell (arrowhead).
