# Supplementary material for: A novel proneural function of Asense is integrated with the sequential actions of Delta-Notch, L’sc and Su(H) to promote the neuroepithelial to neuroblast transition
Source: PLoS Genet. 2023 Oct 23;19(10):e1010991. doi: 10.1371/journal.pgen.1010991 (PMC10621995; doi:10.1371/journal.pgen.1010991)
Supplement: S8 Fig — Confocal images taken at equivalent deep layers in the OPC of control (c855a-Gal4) and c855a-Gal4/UAS-Dl-DN larvae after a 36h induction. Notice that there are not additional L’sc+ cells in the c855a-Gal4>-Dl-DN sample compared to the control. (PDF) [file pgen.1010991.s008.pdf]

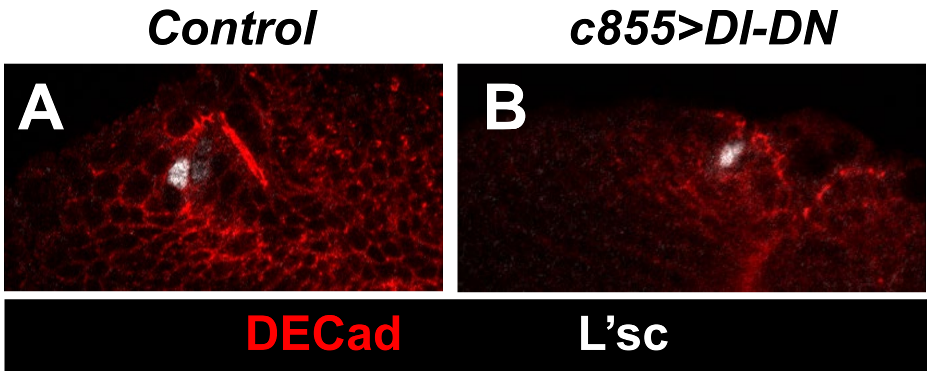

S8 Fig. The downregulation of Notch in the NE does not induce L’sc expression. Confocal images taken at equivalent deep layers in the OPC of control (*c855a Gal4*) and *c855a-Gal4/UAS-Dl-DN* larvae after a 36h induction. Notice that there are not additional L’sc+ cells in the *c855a-Gal4>-Dl-DN* sample compared to the control.
