# Supplementary material for: A novel proneural function of Asense is integrated with the sequential actions of Delta-Notch, L’sc and Su(H) to promote the neuroepithelial to neuroblast transition
Source: PLoS Genet. 2023 Oct 23;19(10):e1010991. doi: 10.1371/journal.pgen.1010991 (PMC10621995; doi:10.1371/journal.pgen.1010991)
Supplement: Attachment — Submitted filename: Response to Reviewers-Rev.pdf [file pgen.1010991.s011.pdf]

POINT BY POINT RESPOSE TO REVIEWERS COMMENTS

Reviewer #1:

This paper from Martin et al. describes a new function of the transcription factor Asense (Ase) during the neurogenesis of the Drosophila optic lobe, particularly in the outer proliferation center (OPC). The main conclusions are that Ase works as a proneural gene promoting the NE-NB transition during OPC development and, on the other hand, there is a novel cell type in the transition between neuroepithelial cells and neuroblasts with the highest Ase expression that the authors called Ase peak cells (PIII in the discussion). Overall, the conclusions are interesting and generally supported by the data. However, some precision needs to be added in the description of the data, use of appropriate statistical analysis, and some additional experiments are required to be sure that the functions assigned to Ase are robust.

Reply: We thank the Reviewer for the comments and criticisms, which we think have helped us to improve the Manuscript. Please, see here below our point by point reply to your comments and how we have addressed them in the revised MS.

Major comments
1. In the time-restricted gene expression modification experiments, authors only use temperature, which is not ideal. Some strong GAL4 lines can also work at a low temperature. Why GAL80ts was not used for these experiments? For instance, it has been used in Egger et al. 2010.

Reply:

As the Reviewer pointed, some GAL4 lines can induce UAS-trangen expression at low temperature. We were aware of this. Thus, as we mentioned in the Material and Methods section (page 7 line 163-5), we set the initial restrictive temp. carefully <17ºC. In those conditions, as we show in Suppl. Fig.2A-D, we found "***no consistent transgene induction or concomitant phenotypes***". Therefore, we trust that the results obtained out of our time-restricted gene expression experiments are correct. Regarding the report by Egger and cols. 2010, we think that they used GAL80ts for the generation of clones. However, in the Material and Method section, they describe their Dl-DN experiments in the following manner: '***'For the dominant-negative Delta experiments we used w; TM6B/TM3, UAS-Dl.DN (Huppert et al., 1997) driven by GAL4c855a***''. Later they added: '***'For dominant-negative Dl experiments, larvae of the genotype w; +/+; TM3, UAS-Dl.DN/GAL4c855a were raised at 25°C''***. Hence, presumably, in their Dl-DN experiment they neither used GAL80ts nor had temperature control over Gal4 activity, in contrast with our protocol. We think that this is very important for the interpretation of results. Hence, as we now emphasize in the Revised MS at the introduction of our timing control experiments (page 13, line 336), having a good timing control is crucial to distinguish between the effects of inductive vs permissive factors/signals. Accordingly, in the Discussion (page 25, line 723-33), contrary to what was concluded in previous reports (for instance, Egger et al, 2010), we favor the idea that the down-regulation of Notch rather than being a final instructive action promoting NB fate, it is an intermediate permissive or priming step that requires additional signaling to proceed.

2. The c820-GAL4 driver is not carefully described, some lower magnification pictures of the

entire optic lobe will allow them to clearly demonstrate that this driver is specific to a subset of OPC cells.

Reply: Following the Reviewer request, we now show lower magnification pictures of the entire optic lobe of c820-GAL4>dGFP and c855a-GAL4> dGFP at superficial and deep levels (Suppl. Figure 1)

3. Since most of the work intends to address ase function, using only one RNAi line is not ideal. Authors should try to test another RNAi line to be sure that part of the observed phenotype is not due to off-target effects.

Reply: Following the Reviewer request we have found (and used) a second UAS-Ase-RNAi line that efficiently downregulates Ase expression at the NE-NB transition and ,moreover, reproduces the phenotypes previously observed in the ase^1 allele and the first RNAi line. These data are presented in the new Suppl-Figure 3.

4. In the same line, why not complement some of the RNAi experiments with MARCM clones or other loss of function ase clones? They already have a mutant of ase that could be recombined with an FRT chromosome

Given the confirmation of the robust phenotype obtained with the second Ase RNAi line, we consider that the use of an ase FRT mutant would not provide new or different information and presumably, presents the problem that only a very small proportion of clones (i.e. only those covering the transition zone) would be useful for the specific purpose of this experiment.

5. Could the authors explain why although L(sc) is not able to promote by itself the NE-NB transition, it still can induce Ase expression in the transition zone

Reply: The Reviewer asks an interesting question that contains an apparent contradiction of our results. Let me reformulate it: Why *L'sc GoF* in the NE is not able to induce NE-NB transition if it is capable of inducing Ase at the transition? The reason behind this resides in two conclusions of our data. First, L'sc promotes *ase* expression in a non-cell autonomous manner and does it through Delta-Notch signaling in the adjacent cells. The second observation that we have made is that Delta-Notch signaling is only sufficient to promote *ase* expression towards the medial side of the transition but not in the lateral side. Thus, as we discussed in the previous MS, *ase* expression seems to be tightly repressed by an unknown mechanisms in most of the NE. We now discuss this in page 24, line 703, ***"our Dl and l'sc GoF data indicate that ase expression is repressed tightly in the NE by an unknown mechanism that possibly serves to prevent its premature upregulation by Notch signaling in the lateral side, ensuring in this manner the medial to lateral direction of the neurogenic wave"***. Accordingly, L'sc GoF clones only promote *ase* at the outside border of the NE (Fig. 5. C,D) Therefore, driving L'sc in the whole NE with c855a-Gal4 is not effective in transforming NE cells into NBs (Fig. 4A,B).

6. Regarding Table I, what is the control condition? What is the statistical test used?

Reply: Following the Reviewer comment, we realized that the way we presented Table I might had been a bit confusing. Table I is in fact a comparative (timing and quantitation) summary of the experiments performed by driving Ase, Dl-DN, L'sc, and now, additionally, Notch RNAi. The phenotype and genetic control (c855a-Gal4) are described in detail in the corresponding main Results text and figures. To avoid confusion to the readers, we now have accordingly explained these points in the Table text and have added a column to direct readers to the corresponding Figures.

7. In Fig2G. What is the control? Just a control strain is not appropriate for an experiment in which a GAL4 is combined with an RNAi line. If the control is correct and three groups are going to be compared, a t-test is not the proper test to use, ANOVA should be used instead.
Reply: The Reviewer is completely right. We apologize for this mistake. These were independent experiments and hence, quantitations should have been shown with the corresponding controls. We have accordingly corrected this and the graph is now shown in Fig.2I

8. Regarding the Notch-dependent mechanism. How do the authors think that Notch promotes L´sc expression usually during the transition? Non-autonomous effect?

Reply: We thank the Reviewer for this question, because we realized that did not make clear the point before. As we remark now in the Results (Page 17, line 462, Fig.8A) we found that L'sc was always found inside N_{ICD} clones. This result can be only explained by a cell-autonomous action as we now state in the Discussion (Page 22, line 616): "***This action appears to work cell-autonomously since L'sc was always induced inside N_{ICD} clones".***

9. In Suppl figure 4E authors use Mann-Whitney test to determine significance but in suppl Fig6, they use t-test instead. Please explain the rationale since is basically the same type of data. This explanation should be added to the methods section.

Reply: Following the Reviewer request we have added the explanation for the differential use of these methods (Page 9, line 222) "***The data was analyzed using Microsoft Excel 2010 and SigmaStat, employing Student t-tests for samples drawn from normally distributed populations with the same variances or alternatively, Mann-Whitney Rank Sum tests for non-normal populations or populations with unequal variances. Differences in both cases were considered significant at P < 0.05"***

Minor comments

1. Please add line numbers, it makes the correction easier

Reply: Following the Reviewer request we have added line numbers

2. Scale bars should be added to every figure.

Reply: As we now explain in more detail in the Methods section (Page 8, line 209) ***"Equal processing was applied to control and mutant images that were selected from equivalent positions in the OL comprising equal digital area size".*** This procedure makes in principle unnecessary to include scale bars to compare the partner samples. Nevertheless, we have included scale bars in the images of those experiments (Figures 3 and 4, Suppl-Figure 6) in which we detected change in the size of the samples after genetic modifications.

3. In the schematics, PI and PII cells are drawn with a rectangular shape in Figure 8, while PIII cells are round, while in Fig1 and Fig6, L´sc cell is round. Most literature agrees that PII cells are more like NE cells with high levels of DCad.

Reply: We thank the Reviewer for pointing to this mistake, which has been accordingly corrected in the new version of those figures.

Reviewer #2

This manuscript by Martin *et al.* (Tejedor's labs) provides new insights into the mechanism underlying the transition from neuroepithelial cell fate to neurogenic cell fate (NE-NB transition) using the *Drosophila* optic lobe as a model system. In particular, they describe in detail the expression of Asense during this transition. Using genetic and cell biology tools, they show that Asense is necessary and sufficient to promote this NE-NB transition. This in itself is interesting because this factor is considered a neural rather than proneural factor. In addition, the authors provide experimental evidence that allows them to propose a mechanism for the NE-NB transition that integrates Asense, the proneural transcription factor L'sc and the Notch signaling pathway. In particular, they propose three transitional cell fates between the NE and the neuroblasts fates.

The data presented are of high quality and well support the conclusions. In particular, some data contradict the current NE-NB transition model and they present compelling evidence to support their conclusions. Moreover, they do not hesitate to discuss the unknown elements of the proposed mechanism. Overall, I consider this work of interest to colleagues working on the processes of cell fate transitions and in particular neurogenesis. While appreciating the quality of the work, interesting and thoroughly executed, I have a few remarks and concerns, which do not change the main conclusion of this work and I am sure that the authors should be able to address.

We are grateful to Dr Gho for the rewarding comments and wise criticisms, which we think have helped us to improve the Manuscript. We have tried to address your concerns as we explain here below point by point.

1. My main concerns relate the role of Su(H) on the NE-NB transition. Su(H) expression has been described using LacZ reporters (Fig 6C, D). Under these condtions, it is not possible to conclude that "Su(H) is absent from NE cells". As such, the idea that **"Notch signals can be transmitted independently of Su(H)" required a more precise evidence**. In particular, evidence obtained from Su(H) null clones. On this regard, the labeling of Figures 6 C and D is incorrect. It is not Su(H) that is detected but a cytoplasmic □-Gal, as a Su(H) reporter.

Reply: Following the Reviewer we have worked in several directions to clarify the issues regarding the role of Su(H) on the NE-NB transition. First, we agree that a reporter can not necessarily reflect the pattern of the protein but possibly only the cells in which the gene is expressed. Accordingly we have made the changes in the labeling of the corresponding panels in Fig. 6.

Unfortunately, the two anti-Su(H) antisera that we tried in the pass did not work well in the larval OL (they gave too dirty and weak signal). In the absence of that, we now tried a third Su(H) reporter, actually a Gal4 line that provided to us a Notch signaling expert lab. Unfortunately, this line showed a preponderant expression in enwrapping glia of the OL, which lack of interest in the context of the present work. Hence, we do not mention it in Results.

We are aware of only a single report of anti-Su(H) immunostaining in the larval brain (Wang et al., 2011). As we now discuss (Page 24, line 692) this exhibited a widespread labeling in the larval brain including most, if not all, OL cells although no genetic controls were used to ensure the specificity of that expression pattern. We also take into consideration in the Discussion, following those lines, the results in the literature about the phenotype of "***Su(H) LoF clones located in the medial side of the NE showed alterations in the NE-NB transition, indicating that Su(H) is possibly required before the proneural wave for a proper transition***". In any case, we think that these data do not contradict our proposal of a key role of Su(H) on downregulating *l'sc* and *ase* expression peaks to end the proneural wave. As we now discuss in Page 24, line 698, this proposal is based on the expression pattern reproduced by the two Su(H)-Lac Z reporters (this MS) and the data reported by Perez-Gomez and cols. 2013 as discussed in Page 24, line 684-91. Most important, this function is fundamentally based on the *Su(H) GoF and RNAI* driven in transitioning cells, which are now shown in the new Fig.7 and Fig.8C-F.

We thank you for the suggestion to look for more precise evidence that Notch signals may be transmitted independently of Su(H). In this regard, we carried out two sets of experiments: we analyzed *Nicd* clones generated in NBs, which showed a repression of Ase expression (see Fig.5K). Conversely, we drove Dl-DN expression in NBs and found that this caused an increase in Ase expression (Fig. 5L-N). Remarkably, these new results are opposite to the respective effects that Notch upregulation and downregulation caused in transitioning cells (Fig. 5E-J). Together, these data let us to conclude (Page 24, line 700***) "The facts that we have found that Notch signaling promotes Ase in the transition but represses it in NBs suggest that Su(H) may help to switch Notch signaling from promoting to repressing ase expression at these transitioning stages (Fig. 9C,D)"***

2. The idea that Su(H) plays multiples roles according to its expression level recalls the situation in mechanosensory organs on the notum. Posakony's lab identified an autoregulatory enhancer on the Su(H) promotor required for a high expression level on late sensory organs (Barolo et al. 2000). It will be interesting to test wheter this enhacer (ASE5) controls Su(H) expression in the optical lobe, particularly in the NB area.
Reply: We appreciate very much your advice. Investigating the regulation of Su(H) expression in the OL is a very interesting goal and, the *Su(H) (ASE5)* could be possibly a suitable tool to try in the OL. Unfortunately, given the multiple interactions of signaling pathway at the NE-NB transition, we can not approach this objective within the scope of this MS".

3. Recently, Doe's group (Dillon et al 2022) published an RNA-seq analysis of a single larval brain cell. Is it possible to link the different cell types described by Doe to those identified by immunodetection, this work? Such a comparison can reveal whether the mechanism driving the NE-NB transition is transcriptional or post-transcriptional based.

Reply: The Single cell RNA-seq analysis reported by Dillon and cols. is a great work that intends to find the transcriptional finger print of optic lobe cells. This allowed them to identify progenitor families that change gene expression along development, This work has been very useful to identify cell type specific genes and to propose differentiation trajectories of neurons Unfortunately, the fact that progenitors at the NE-NB transition are very much under-represented because of their short life (for instance we find less than 50 Ase peak cells per hemisphere in third instar larvae) makes very difficult to use this method to study them in order to answer the quite interesting question of whether the mechanisms driving the NE-NB transition are transcriptionally or post-transcriptionally based.

4. The expression of Ase is shown in detail in Figure 1. In this figure, two optical planes are shown. One tangential to the surface (surface layer) and another perpendicular to the surface (deep layer). These data are convincing. However, the authors only show a deep view for the data regarding the expression of different markers in a context of loss of function

for *ase* (Figure 2). I think these data will be much more convincing if a surface view is also displayed (as shown in Figure 3 for the gain of function for *ase*). Also, it will be more informative for me to have a surface and a depth view of the expression pattern of the Gal4 drivers c855a and c820 (Suppl. Figure 1). Same commentary for Figures 5E and F.
Reply: As you requested, we now also show surface layer confocal images of the mentioned experiments in the corresponding Figures, additionally including the c855a and c820 expression patterns (Suppl. Figure 1).

5. In Figure 3E is shown a clone of cells overexpressing Ase. The authors indicate that this experiment attempts to assess whether Ase promotes the formation of NBs (Mira-expressing cells) in a cell-autonomous or non-cellular manner. I do not see the analysis of this experiment: number of Mira positive cells at the border against those within the clones. In any case, they do not conclude whether Ase acts cell autonomously or non-cell autonomously. In short, I do not see the point raised.
Reply: We think that your point is correct. Following your concern, we revised all the Ase GoF clones of that experiment and, as we now state in (Page 12, line 305) "***none of these clones exhibited ectopic Mira at their edge***" These results favor the idea of a cell-autonomous action as we now suggest in the same paragraph.

6. This work is mainly based on analyzes of fixed tissues. It is therefore difficult to draw conclusions on the temporality of the events studied. I think that authors should be more careful when concluding on the effects on the dynamics of the processes studied. Or clearly explain that in this system it is possible to conclude about the dynamics of processes studying fixed tissues. This criticism concerns sentences like:

"The down-regulation of Notch induces the NE-NB transition at a slower rate than the gain of function of *ase*"

"Together these experiments demonstrate that the GoF of *ase* is capable of inducing a more rapid and efficient NE-NB transition than the down-regulation of Notch."

Reply: We partially agree with this concern. Performing a detailed analysis of the dynamics of the cellular processes induced by GoF or RNAi experiments using fixed tissue is not possible and this was not our goal. As we now introduce the Results section, our timing control experiments were intended to distinguish between the effects of inductive vs permissive actions of the factors/signals (page 13, line 336), "***In order to distinguish between inductive and permissive actions, we decided to compare the time course of the phenotypic changes induced by inhibiting Notch in the NE to that of ase GoF. We assumed that an inductive factor/signal should produce an immediate effect (i.e. upregulation of NB markers) in NE cells. In contrast, a permissive factor would prime the cells until they are reached by the inductive signal at the proper position of the neurogenic wave and, consequently, these concomitant actions should require longer time to produce the effect''.***

Our results (summarized in Table I) clearly show how in the short term only Ase is capable of inducing the expression of NBs marker in NE cells compared to L'sc and Dl-DN and N RNAi. It is important to emphasize that Dl-DN efficiently downregulates Notch in the short term (see Suppl. Fig.5 A,B). All the genetic modifications (Ase GoF, L'sc GoF, N RNAi and Dl-DN) induced the transformation of NE into NBs in the long term although with different efficiency. The clear difference between the effects at the short and the long term is what allows us to conclude in a straightforward manner that Ase is quicker and more efficient than the others. These results, together with some data from literature, allow us later to Discuss and propose in Page 25, line 729: "***Together, these data strongly suggest that the down-regulation of Notch rather than being a final instructive action promoting NB fate, it is an intermediate permissive or priming step that requires additional signaling to proceed.".***

7. The model that they propose (Figure 8) is in according with the data shown. It is not clear to me how this model is consistent with the fact that the NE-NB transition is a wave that sweeps from medial to lateral on the neuroepithelium. Could the authors explain how the model proposed is consistent with a wave that progresses on the tissue?
Reply: Following your suggestion, we have modified the model to a step by step format (now in Fig.9) that hopefully explains better how we think the NE-NB transition progresses from medial to lateral on the neuroepithelium. Additionally, we discuss in several places how the mechanisms that we propose may interact with other signaling pathways.

Minor points:

The antibodies directed against PatJ and β-Gal are not referenced in the Immunohistochemistry paragraph of the Materials and Methods.
Reply: Thank you for detecting the omissions. These have been now corrected.

The labels in figure suppl-Figure 3 are wrong. Surface view (A,C) and deep layers (B,D).
Reply: Thanks for detecting the mistake. This has been now corrected.

Reviewer #3:

This study investigates the proneural role of various bHLH genes (l'sc, ase and Su(H)) downstream to Notch during the neuroepithelium-to-neuroblast (NE-to-NB) transition in the optic lobe of the Drosophila larval brain. In the larval optic lobe, a differentiation (or proneural) wave progressively converts the proliferative neuroepithelium into asymmetrically dividing neuroblasts. Various pathways have been shown to have key roles in the regulation of this wave such as the EGFR, JAK/STAT, Notch or ecdysone pathways. While this process has been actively investigated over the last decade, it is not completely understood. In particular, the dynamics of Notch signalling and the precise mechanisms by which Notch promotes or inhibits the proneural wave remain unclear. Here the authors investigate in details the sequential expression of Su(H), ase and l'sc along the progressive NE-to-NB conversion. They clearly show that the fine NE-to-NB transition involves 4 states defined by the sequential expression of E(spl), L'sc, Ase and Su(H). They propose that Ase can have a proneural role and that Su(H) is required in NBs to down-regulate Ase. Finally, they propose that these transitions are at least partly regulated by the Notch pathway in an unconventional manner, when compared to the classical lateral inhibition model.

The authors only focus on the Notch pathway and its interactions with bHLH genes, while other studies have shown that **other pathways such as the EGFR and JAK/STAT can also interact with the Notch pathway to regulate its targets**. Consequently, several of the conclusions somehow contradict the study of several labs (e.g. Tabata/Yasugi, Sato, Brand). There is therefore a need to clarify this situation in the discussion to demonstrate that this study brings a new and pertinent angle to our understanding of the mechanisms that drive the NE-NB transition.

Reply: We thank the Reviewer for the comments and criticisms, which we think have helped us to improve the Manuscript. As it can be followed from our point by point reply here below, we have tried to fill the possible gaps and address the concerns raised by the Reviewer.

1. More precisely:- The authors mention that "GoF of ase is capable of inducing a more rapid and efficient NE-NB transition than the down-regulation of Notch". It is unclear to me how relevant it is to compare the dynamics of a GOF and LOF experiments in such a complex

system, especially when using mis-expression of a dominant negative form of Delta which may not lead to a complete knockout. Is the dynamics the same in Notch-RNAi conditions. Lastly, I am not sure to understand why this argument is necessary? Can you be more explicit about that?

Reply: As we now introduce in the Results section, our timing control experiments were intended to distinguish between the effects of inductive vs. permissive actions of those factors/signals (page 13, line 336).``***In order to distinguish between inductive and permissive actions, we decided to compare the time course of the phenotypic changes induced by inhibiting Notch in the NE to that of ase GoF. We assumed that an inductive factor/signal should produce an immediate effect (i.e. upregulation of NB markers) in NE cells. In contrast, a permissive factor would prime the cells until they are reached by the inductive signal at the proper position of the neurogenic wave and, consequently, these concomitant actions should require longer time to produce the effect``.*** Our results (summarized in Table I) clearly show how in the short term only Ase is capable of inducing the expression of NB markers in NE cells compared to L'sc and Dl-DN. It is important to emphasize, as we explain now in Page 13, line 343 that the Dl-DN knockdown was intended "***To emulate the down-regulation of Notch protein that takes place at the proneural wave front (Wang et al., 2021)***. Also we should remark that at the NE-NB transition there is only a partial down regulation of Notch protein and that our results show that Dl-DN efficiently downregulates Notch in the short term (see Suppl. Fig.5 A,B). All the genetic modifications (Ase GoF, L'sc GoF, N RNAi and Dl-DN) induced the transformation of NE into NBs in the long term although with different efficiently. The clear difference between the effects at the short and the long term is what allows us to conclude in a straightforward manner that Ase is quicker and more efficient than the other factors/signals. These results, together with some data from literature, allow us later to Discuss and propose in Page 25, line 729. "***Together, these data strongly suggest that the down-regulation of Notch rather than being a final instructive action promoting NB fate, it is an intermediate permissive or priming step that requires additional signaling to proceed".***

Following the Reviewer request, we have performed N RNAi in the NE under the same timing and conditions. The results are depicted in the new Suppl. Fig.6 (and compared in Table I). As described in Page 14, line 353 "***N RNAi induced for a short term with the c855a-Gal4 driver did not cause any detectable downregulation of Notch or alterations in the NE-NB transition*** (Suppl. Fig. 6A,B; Table I). This result was somehow expected for us (and the reason why we did not used previously N RNAi for a short term effect) since in our experience RNAi needs in most cases, at least 24h to produce enough protein downregulation. In any case, a we state in Page 14 `***Nevertheless, the depletion of Notch in the NE throughout the neurogenic period in the OPC (36h) using Dl-DN and N RNAi caused extensive co-expression of NE and NB markers, and a concomitant reduction in the NE area (Fig. 4D,E; Suppl. Fig. 6A,C; Table I)*** . Hence, at the end, N RNAi cause equal effect as Dl-DN. We hope these data clarify the issue.

- The authors proposes that L'sc expression is promoted by Notch signaling. This is contrary to what is observed during lateral inhibition where N represses L'sc. **The authors interpret this result as a "non-conventional effect", but they don't provide any explanation on the underlying mechanism**. This interpretation differs from various studies performed by Yasugi and Sato:
1: Sato M, Yasugi T, Minami Y, Miura T, Nagayama M. Notch-mediated lateral inhibition regulates proneural wave propagation when combined with EGF-mediated reaction diffusion. Proc Natl Acad Sci U S A. 2016 Aug 30;113(35):E5153-62. doi:10.1073/pnas.1602739113. Epub 2016 Aug 17. PMID: 27535937; PMCID: PMC5024646.
2: Yasugi T, Sugie A, Umetsu D, Tabata T. Coordinated sequential action of EGFR and Notch signaling pathways regulates proneural wave progression in the Drosophila optic lobe. Development. 2010 Oct;137(19):3193-203. doi:10.1242/dev.048058. Epub 2010 Aug 19.

PMID:
These previous studies convincingly demonstrate using genetic experiments and mathematical modelling that l'sc is activated by EGFR signalling and failed to be activated by Notch signaling when EGFR signalling is down-regulated consistent with a pre-existing lateral-inhibition mechanism.

In the present manuscript, the **authors do not take in account the potential role of EGFR to interpret their results**, therefore it is unclear how this work integrates with the previous findings. It also makes it difficult to understand where the novelty is compared to these previous studies. This should be clarified

Reply: We agree with the Reviewer that other signaling pathways, particularly EGFR, play key roles in the NE-NB transition. As a matter of fact, we made this point clear in the Introduction of the MS (now in page 5, line 87). "***it requires the coordinated action of several signaling pathways, mainly JAK/STAT, EGFR, Fat-Hippo and Notch"***.

We were in principle reluctant to speculate with the involvement of other pathways in the interpretation of our working model as we did not perform experiments to address this matter. Nevertheless, following the Reviewer request, we have now tried to clarify how our results could be integrated with previous findings of other labs, particularly, regarding the role of EGFR signaling. We think that there is compelling evidence for the cooperation of EGFR and Notch signaling in the regulation of L'sc expression at the NE-NB transition. However, our data indicates that the initial action in triggering that expression is driven by Notch signaling in contrast to the conclusion of Yasugi and cols, 2010. We also disagree with the idea that activation of L'sc requires "***a pre-existing lateral-inhibition mechanism***" as brought by previous papers and pointed by the Reviewer. Thus, in page 22, line 629 we discuss this matter based on our results and the interpretation we made out of other researchers data: "***It has been clearly shown that EGFR signaling is activated in the most medial NE and transitional cells of the OPC and, accordingly, it was proposed that this signal induces L'sc expression (Yasugi et al., 2010). Nevertheless, the ectopic activation of EGFR signaling in clones resulted in a lateral shift of the L'sc expression peak respect to the surrounding wt tissue (Yasugi et al., 2010) but not in the upregulation of l'sc within most, if not all, the clone area, as we have seen by the strong activation of Notch signaling with N_{ICD}. Interestingly, overexpression of N^{act} resulted in the expression of PntP1, a major transducer of EGFR signaling (Gabay et al.,1996), in NE cells (Weng et al., 2012; Yasugi et al., 2010). Furthermore, the overexpression of N^{act}, in pnt LoF clones covering the NE-NB transition zone resulted in the lack of L'sc expression (Yasugi et al., 2010). These data suggest that the upregulation of l'sc by Notch signaling may require the priming activation of EGFR signaling Noteworthy, the coactivation of Notch and EGFR signaling in clones seems to produce the same effect in the transition as the activation of Notch alone since the cells remain expressing L'sc and do not progress to NB differentiation (Yasugui et al., 2010).*** We hope these data clarify the issue.

Otherpoints:
- Flybase indicates that the usual protein names of shg are E-Cadherin (E-Cad or ECad) or DE-Cad (for Drosophila ECad) but not DCad. It would be nice to stick to one of the previously used names.

Reply: Following the Reviewer suggestion, we now have used DECad all along the manuscript.

- The ase-RNAi line does not seem to very efficiently knockdown Ase, and **the phenotype is hard to notice on the figure**. It may be wise to test other RNAi lines and check whether phenotypes are more convincing.

Reply: As we explain in page 11, line 275 the purpose of the Ase RNAi experiment in the way we performed it, was not to eliminate Ase expression but to assess ***" whether the peak of Ase expression is required for the correct NE-NB transition"*** by driving *ase-RNAi* in the most medial NE and transition zone cells using the *c820-Gal4* line. We think that the downregulation of the Ase peak is good enough and the phenotype of coexpression of NB and NE marker quite clear. Moreover, we have quantified it in Fig. 2I. Nevertheless, we have now added surface layer views (Fig. 2E,F) that may improve the visualization of the phenotype. Furthermore, following the Reviewer suggestion, we have used a second UAS-Ase-RNAi line, which efficiently downregulates Ase expression at the NE-NB transition and ,moreover, reproduces the phenotypes previously observed in the *ase1* allele and the first RNAi line. These data are presented in the new Suppl-Figure 3

- In some panels, the genotype is indicated, in some it is not (e.g. Fig6). Please. homogenise.

Reply: we did not indicate genotype in Fig.6 as well as Fig.1 because these are descriptive immunostainings carried out in wt brains as indicated in the legends. We have revised the rest of figures to make sure that genotypes are mentioned in the panels when the space allows it. Otherwise, they have been mentioned in the legends.

- Figure 5I-L should be moved to Figure 6

Reply: We, agree with the Reviewer that moving the results of Su(H) LoF and GoF to a later position in the MS is convenient for the flow of information. Nevertheles, we consider that Fig.6 is not a convenient place as it contains just a description of expression patterns of Notch signaling elements. Thus, we decided to move those results to an independent figure (Fig.7) in which now we have also included the graph (quantitative data) that was previously displayed in Suppl. Fig.6

- In some figures, clones are sometimes indicated as "clon" (e.g. Fig7). Please correct.

Reply: These have been accordingly corrected.
