# Supplementary material for: A novel proneural function of Asense is integrated with the sequential actions of Delta-Notch, L’sc and Su(H) to promote the neuroepithelial to neuroblast transition
Source: PLoS Genet. 2023 Oct 23;19(10):e1010991. doi: 10.1371/journal.pgen.1010991 (PMC10621995; doi:10.1371/journal.pgen.1010991)
Supplement: Attachment — Submitted filename: Review Martins MS.docx [file pgen.1010991.s010.docx]

This manuscript by Martin *et al.* (Tejedor's labs) provides new insights into the mechanism underlying the transition from neuroepithelial cell fate to neurogenic cell fate (NE-NB transition) using the *Drosophila* optic lobe as a model system. In particular, they describe in detail the expression of Asense during this transition. Using genetic and cell biology tools, they show that Asense is necessary and sufficient to promote this NE-NB transition. This in itself is interesting because this factor is considered a neural rather than proneural factor. In addition, the authors provide experimental evidence that allows them to propose a mechanism for the NE-NB transition that integrates Asense, the proneural transcription factor L'sc and the Notch signaling pathway. In particular, they propose three transitional cell fates between the NE and the neuroblasts fates.

The data presented are of high quality and well support the conclusions. In particular, some data contradict the current NE-NB transition model and they present compelling evidence to support their conclusions. Moreover, they do not hesitate to discuss the unknown elements of the proposed mechanism. Overall, I consider this work of interest to colleagues working on the processes of cell fate transitions and in particular neurogenesis. While appreciating the quality of the work, interesting and thoroughly executed, I have a few remarks and concerns, which do not change the main conclusion of this work and I am sure that the authors should be able to address.

_______

My main concerns relate the role of Su(H) on the NE-NB transition. Su(H) expression has been described using LacZ reporters (Fig 6C, D). Under these condtions, it is not possible to conclude that "Su(H) is absent from NE cells". As such, the idea that "Notch signals can be transmitted independently of Su(H)" required a more precise evidence. In particular, evidence obtained from Su(H) null clones. On this regard, the labeling of Figures 6 C and D is incorrect. It is not Su(H) that is detected but a cytoplasmic β-Gal, as a Su(H) reporter. The idea that Su(H) plays multiples roles according to its expression level recalls the situation in mechanosensory organs on the notum. Posakony's lab identified an autoregulatory enhancer on the Su(H) promotor required for a high expression level on late sensory organs (Barolo et al. 2000). It will be interesting to test wheter this enhacer (ASE5) controls Su(H) expression in the optical lobe, particularly in the NB area.

Recently, Doe's group (Dillon et al 2022) published an RNA-seq analysis of a single larval brain cell. Is it possible to link the different cell types described by Doe to those identified by immunodetection, this work? Such a comparison can reveal whether the mechanism driving the NE-NB transition is transcriptional or post-transcriptional based.

The expression of Ase is shown in detail in Figure 1. In this figure, two optical planes are shown. One tangential to the surface (surface layer) and another perpendicular to the surface (deep layer). These data are convincing. However, the authors only show a deep view for the data regarding the expression of different markers in a context of loss of function for *ase* (Figure 2). I think these data will be much more convincing if a surface view is also displayed (as shown in Figure 3 for the gain of function for *ase*). Also, it will be more informative for me to have a surface and a depth view of the expression pattern of the Gal4 drivers c855a and c820 (Suppl. Figure 1). Same commentary for Figures 5E and F.

In Figure 3E is shown a clone of cells overexpressing Ase. The authors indicate that this experiment attempts to assess whether Ase promotes the formation of NBs (Mira-expressing cells) in a cell-autonomous or non-cellular manner. I do not see the analysis of this experiment: number of Mira positive cells at the border against those within the clones. In any case, they do not conclude whether Ase acts cell autonomously or non-cell autonomously. In short, I do not see the point raised.

This work is mainly based on analyzes of fixed tissues. It is therefore difficult to draw conclusions on the temporality of the events studied. I think that authors should be more careful when concluding on the effects on the dynamics of the processes studied. Or clearly explain that in this system it is possible to conclude about the dynamics of processes studying fixed tissues. This criticism concerns sentences like:

“The down-regulation of Notch induces the NE-NB transition at a slower rate than

the gain of function of *ase*”

“Together these experiments demonstrate that the GoF of *ase* is capable of inducing a more rapid and efficient NE-NB transition than the down-regulation of Notch.”

The model that they propose (Figure 8) is in according with the data shown. It is not clear to me how this model is consistent with the fact that the NE-NB transition is a wave that sweeps from medial to lateral on the neuroepithelium. Could the authors explain how the model proposed is consistent with a wave that progresses on the tissue?

Minor points:

The antibodies directed against PatJ and β-Gal are not referenced in the Immunohistochemistry paragraph of the Materials and Methods.

The labels in figure suppl-Figure 3 are wrong. Surface view (A,C) and deep layers (B,D).
